# Effectors of plants pathogenic fungi and fungal like microbes: a comprehensive review on mechanisms, roles, and host interactions

**DOI:** 10.3389/fpls.2025.1626960

**Published:** 2025-07-29

**Authors:** Kainat, Majid Mujtaba, Yuting Wang, Boru Zhou

**Affiliations:** ^1^ State Key Laboratory of Tree Genetics and Breeding, Northeast Forestry University, Harbin, China; ^2^ Key Laboratory of Alien Forest Detection and Control- Heilongjiang Province, College of Northeast Forestry University, Harbin, China

**Keywords:** effectors, fungi, apoplastic, cytoplasmic, plant immunity, phytohormone

## Abstract

Plant ecosystems face primary threats from biological invasions in combination with microbial pathogens whose main threats derive from fungal pathogens. Fungi are essential in maintaining ecological balance by decomposing wood and eliminating weakened trees, but pathogenic fungi can cause devastating effects. This review summarizes the effects of forest pathogenic fungal effectors by evaluating their types, functions, and unique characteristics, along with their impact on host immune response mechanisms. Pathogens attack plants through specific infection strategies that involve effectors to suppress host defense responses and metabolic activities. Plants falling victim to fungal effectors through their interaction with pathogens lose control of key cellular processes that allow the infection to develop. Effectors are categorized into apoplastic and cytoplasmic types, which influence plant immunity through alterations in immune responses. The infection entry process involves microorganisms that release protein effectors as structural and functional modifiers for target cells. The diversity of effectors jointly with their evolutionary processes depends on multiple factors encompassing amino acid content and foundational genomic zones together with interaction period with hosts. Effectors further manipulate phytohormone pathways such as jasmonic acid, ethylene, and salicylic acid to suppress immunity, promote pathogen survival, and establish parasitic compatibility. However, fungal effectors are central to pathogenesis, as they critically redefine plant-pathogen interactions by targeting host defense mechanism, enabling colonization, and driving diseases development. The review evaluates fungal effectors as dual agents which disrupt plant immunity while serving as research tools to study host biology. Exploring effector-mediated mechanisms helps researchers better understand fungal pathogenicity characteristics alongside plant host defense mechanisms. Future inquiries should examine pathway plasticity in effectors across taxonomic domains to better understand fungal pathogenesis in forest ecosystems worldwide.

## 1 Introduction

Since the emergence of plant-pathogen interactions, a relentless co-evolutionary arms race has unfolded between pathogens and their plant hosts (Zhang et al., 2022). Plant pathogens represent a diverse collection of organisms that possess the capability to infect their hosts. Pathogens, ranging from fungi, bacteria, and nematodes to parasitic plants, exploit host resources for survival and reproduction. Pathogens that can infect seemingly healthy plants are known as primary pathogens. Secondary pathogens opportunistically colonize tissues weakened by prior infection or stress (Termorshuizen, 2017). Pathogenic microorganisms extract nutrients from host cells for survival and reproduction, whereas host plants utilize numerous defense mechanisms to restrict pathogen proliferation (Ngou et al., 2022). Among these adversaries, fungal pathogens constitute the predominant category of plant pathogens, whereas other significant plant pathogens encompass bacteria, protists, chromists, nematodes, and some plants (Termorshuizen, 2017). Currently, about 10,000 fungal species are recognized as pathogens of plants (Agrios, 2005; Koeck et al., 2011). Fungi have endophytic, parasitic, saprotrophic or mutualistic relationships with plants (Jayawardena et al., 2018; Zeilinger et al., 2016). Fungal plant pathogens potentially incite devastating ecological and economic damage to agriculture and forestry, and can also cause severe damage to natural ecosystems (Fisher et al., 2012; Hyde et al., 2018; Jayawardena et al., 2021). The development of plant disease is a result of the tripartite interaction of host, pathogen and environment (Jayawardena et al., 2021). Fungal plant pathogens establish compatibility with the host through various ways (Marcato et al., 2017). Fungi can (i) evade detection by the plant monitoring system (Pel and Pieterse, 2013), (ii) release effector molecules capable of suppressing or manipulating the host defense mechanisms (Doehlemann and Hemetsberger, 2013; Dou and Zhou, 2012), (iii) counteract the antifungal chemicals generated by their host plants (Osbourn, 1999). All these processes may be employed concurrently or sequentially throughout the plant-fungus interaction, together facilitating a successful infection (Marcato et al., 2017). The outcome of these interactions hinges a delicate balance influenced by the host, pathogen, and environmental conditions, setting the stage for a complex molecular arms race.

To counter pathogen attacks, plants are capable of identifying microbes through pattern recognition receptors (PRRs) in the host cells. Upon recognizing the adversary, these receptors initiate effective immune responses in the invaded tissue (Pel and Pieterse, 2013). Microbial recognition is facilitated by conserved structures known as microbe-associated molecular patterns (MAMPs) (Pel and Pieterse, 2013). Furthermore, host-derived signals produced during pathogen infection or mechanical injury, known as damage-associated molecular patterns (DAMPs), represent an additional mechanism by which the host detects pathogen invasion (D'Ovidio et al., 2004; Dou and Zhou, 2012; Huffaker et al., 2006). Receptors that detect pathogen associated molecular patterns (PAMPs) and damage-associated molecular patterns (DAMPs) are commonly referred to as pattern recognition receptors (PRRs). In contrast to animal pattern recognition receptors (PRRs), which include both plasma membrane-localized Toll-like receptors (TLRs) and cytoplasmic NOD-like receptors (NLRs), plant PRRs are only composed of plasma membrane-localized receptor-like kinases or receptor-like proteins (Monaghan and Zipfel, 2012). Plants possess a substantial quantity of NLR proteins; yet, they seemingly do not identify PAMPs or DAMPs (Dou and Zhou, 2012). In contrast, NLRs solely identify intracellular pathogen effector proteins and initiate immune responses with high specificity. However, pathogens continually adapt, secreting effector proteins that manipulate host cells to take their advantage (Tsuda and Katagiri, 2010). These effectors can disrupt host metabolism, suppress immune responses, or alter gene expression, depending on whether they act in apoplast (extracellular) or cytoplasm (intracellular).

Effector proteins are secreted by pathogens to manipulate the host to their advantage (Sperschneider et al., 2015). Effectors are molecules that modify the structure and function of host cells, thereby facilitating infection (virulence factors or toxins) and/or initiating defense responses (avirulence factors: Avr). These proteins can be divided into two types according to their target sites in the host plant (Selin et al., 2016). Apoplastic effectors are secreted into the plant apoplast, where extracellular effectors engage with extracellular targets including surface receptors, while cytoplasmic effectors are translocated into the plant cell (Selin et al., 2016). Efficient transport of effectors to the plant is essential for the infection process, irrespective of the effector type. Pathogenic fungi have evolved unique lifestyles and consequently created various effector delivery systems during infection (Selin et al., 2016). The effector proteins are able to alter protein transcription or modify the metabolic pathways of the host cell thereby enhancing pathogenicity (Salehzadeh and Dehghanpour Farashah, 2019). A key unresolved question in plant pathology is how eukaryotic pathogens, particularly (fungi), manipulate host processes to promote infection, highlighting the need for deeper mechanistic insights into these interactions (Whisson et al., 2007).

### 1.2 Plant immune defense mechanisms: MTI and ETI in pathogen resistance

Plants operate with two operational components in their intrinsic defense system through MAMP-triggered immunity (MTI) and effector-triggered immunity (ETI) to combat pathogens (Dodds and Rathjen, 2010; Jones and Dangl, 2006). Each microbe renders MAMPs that function as activating agents through their universally shared molecular signatures that stem from bacterial flagella and fungal chitin alike. MAMPs function as detectable markers that trigger transmembrane pattern recognition receptors (PRRs) present in plant apoplast tissue (Hemetsberger et al., 2015; Irieda et al., 2019). The investigation of plant–pathogen interactions led to substantial advancements in our comprehension of effectors. Harold Flor established the concept of “avirulence factors” in the 1940s because these proteins act as “Avr” factors that activate defense mechanisms by connecting with “R” proteins within plant cells according to his later definitions (Todd et al., 2022b). Resistance proteins primarily group within nucleotide binding (NB) along with leucine rich repeat (LRR) domain (NB-LRR) protein family (Eitas and Dangl, 2010). Disease-limiting processes develop from the biological interaction between Avr proteins and “R” resistance proteins generating conditions hostile to pathogens. The ‘gene-for-gene hypothesis’ demonstrates its origins through how Avr proteins identify and interact with “R” resistance genes. During his research of the flax pathogen *Melampsora lini* and its host plant *Linum usitatissimum* Flor employed this theory. Scientists applied the term “effectors” to label these molecules while recognizing their virulence or avirulence properties based on the resistive genes located in the host plants. Different behavioral functions of effectors determine the effectiveness of pathogens and their ability to cause disease in plant hosts (Win et al., 2012).

The effector-triggered immunity (ETI) activation by viral resistance genes (VRS) creates defense-mediated necrotic lesions which are known as hypersensitive responses. The recognition of pathogen-containing Avr proteins (avirulence proteins) through the plant resistance system allows it to trigger its protective defensive system (ETI). Each Avr protein maintains its primary activity related to virulence because pathogen effectors normally bring advantages to their host although these activities stop working after plant resistance proteins detect them (Bent and Mackey, 2007). The defense system of the host becomes activated through signals produced by MAMPs and effectors. Microbe-associated molecular pattern-triggered immunity (MTI) functions independently from effector-triggered immunity (ETI) as complete defense stages under the plant immunity model named zig-zag (Jones and Dangl, 2006). Plant receptors detecting MAMPs initiate signaling pathways that result in both callose deposition and mitogen-activated protein kinase (MAPK) signaling as well as pathogenicity-related protein expression ultimately producing an oxidative burst with reactive oxygen species (ROS) in plant cells (Chisholm et al., 2006). The response works as a barrier to reduce pathogen spread so plants survive through this MTI defense mechanism. The activity of pathogen-secreted effectors causes MTI disruption which results in what scientists call effector-triggered susceptibility (ETS). ETI manifests with an oxidative burst that causes defense proteins to increase until it leads to programmed cell death (HR) producing phytoalexins which block pathogen dissemination in the infected tissue. Resistant proteins present in plants detect Avr molecules which activates the immune response named effector-triggered immunity (ETI) (Yuan et al., 2021).

## 2 The roles of fungal effectors in host-pathogen interactions

**Figure 1:**
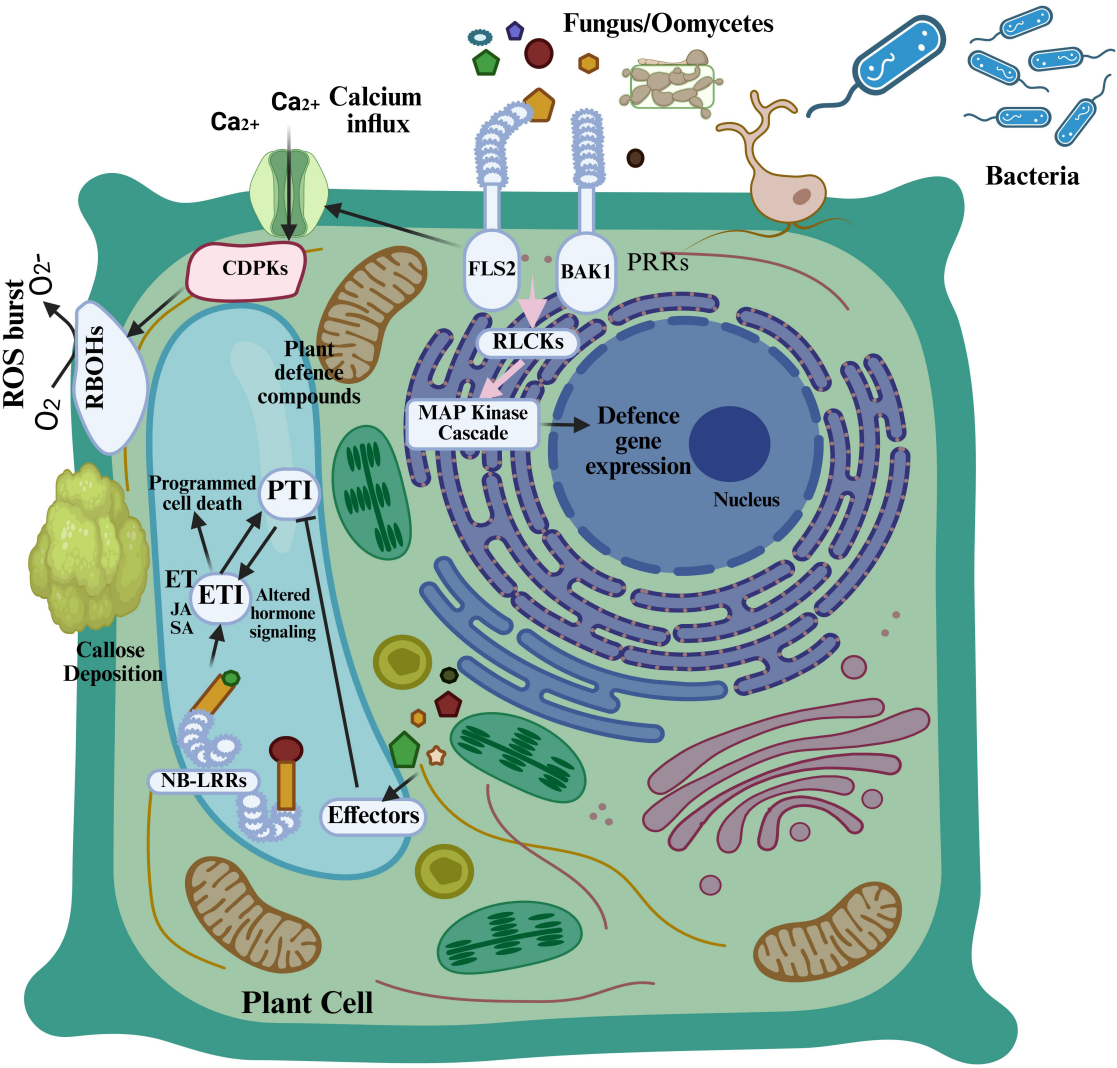
Plant immune system. Pattern recognition receptors (PRRs) in plant cells identify pathogen-associated molecular patterns (PAMPs) and initiate the primary layer of plant immune response, known as pattern-triggered immunity (PTI), which elicits responses including calcium influx and a burst of reactive oxygen species. Pathogens generate effectors to suppress pattern-triggered immunity (PTI), but plant resistance (R) proteins, including NB-LRR proteins, are activated by these effectors to initiate a secondary immune response known as effector-triggered immunity (ETI), which counteracts effectors and induces cell death (Zhang et al., 2022).

Many researchers have examined the ways in which host-pathogen relationships are impacted by the evolutionary conflict between plants and diseases (Langin et al., 2020). Effectors are necessary for plant disease development because they are key players in the induction of susceptibility. Plants build immunological receptors by natural selection with pathogens to detect particular effectors that activate defense responses. The outcome between diseases depends on the continuous interaction between effectors and the host immune system (Lovelace et al., 2023). Understanding the molecular basis of this interaction provides essential knowledge to determine viral disease principles which will guide resistance development. The characterization of particular pathogen-driven effectors has been the major target of scientific research during multiple years (Lovelace et al., 2023). The use of genetic methods has discovered pathogenicity or virulence factors through which scientists gained better insights into the functioning of host–pathogen relationships. Plants have evolved resistance (R) gene-encoding proteins that combat pathogen effectors to initiate immune defense known as effector-triggered immunity ETI (**Figure 1**) (Wang et al., 2015). The primary defense mechanism of plants involves pattern recognition receptors (PRRs) that detect pathogen-associated molecular patterns (PAMPs) to initiate pattern-triggered immunity (PTI) responses, thereby preventing pathogen proliferation (Zhang et al., 2022).

### 2.1 Fungal pathogen ingress and feeding

**Figure 2:**
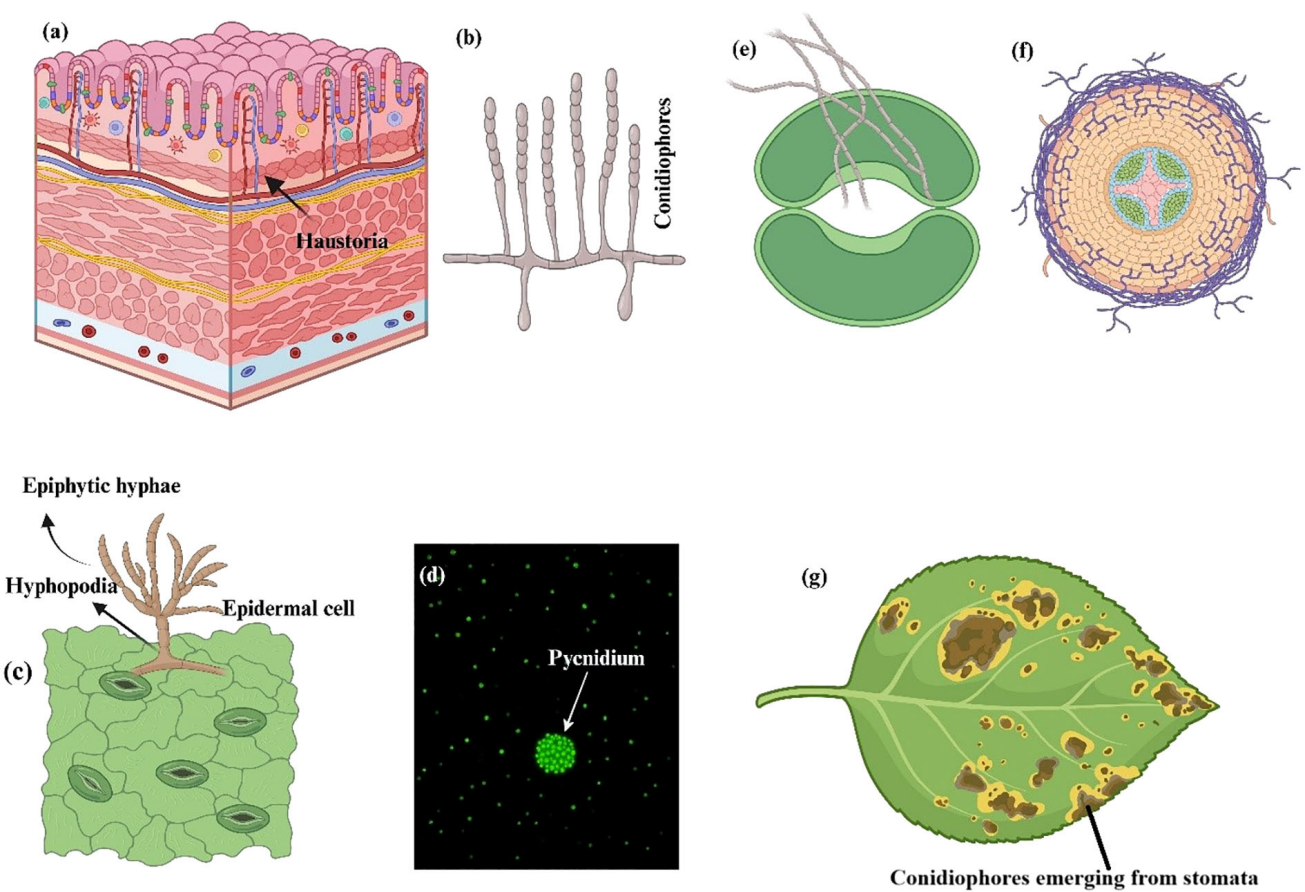
Typical reproductive, feeding, and penetration patterns linked to three different fungal infections. **(a, b)** Barley powdery mildew, *Blumeria graminis f.sp. hordei*. **(a)** Finger-like haustoria in the epidermal cell and epiphytic hyphae piercing the epidermis. **(b)** Conidiophores that produce a lot of conidia. **(c, d)** *Zymoseptoria tritici* with wheat *Septoria nodorum* blotch. **(c)** Epiphytic hyphae that enter through stomata or hyphopodia (arrows). **(d)** A strain that expresses a green fluorescent protein under epifluorescence. The arrow depicts a pycnidium with a large number of conidia. **(e–g)** Tomato leaf mold, *Cladosporium fulvum*. **(e)** Penetration of a stoma by adventitious (runner) hyphae. **(f)** Growth of hyphae around tomato mesophyll cells. **(g)** Conidiophores bearing abundant conidia on the underside of an otherwise healthy tomato leaflet (De Wit et al., 2016).

Pathogenic fungi infiltrate plants by natural apertures (e.g., stomata) or injuries, or they directly penetrate utilizing a penetration peg generated by an appressorium (**Figure 2**). The principal characteristic of biotrophic pathogens is the haustorium, a specialized organ for nutrient acquisition (De Wit et al., 2016). Haustoria form through the local invasion of the cell wall and the invagination of the plasma membrane, encased by an extrahaustorial matrix that facilitates nutrient absorption and communication with the host (Voegele et al., 2001). In powdery mildews, haustoria develop directly from the appressoria within epidermal cells. The rusts and smuts penetrate via stomata and subsequently generate an intercellular mycelium that envelops mesophyll cells, from which a haustorium mother cell and eventually a haustorium are formed. A variety of pathogenic tactics are employed by hemibiotrophic pathogens. Some, including *Magnaporthe oryzae* and *Colletotrichum* spp., create intracellular feeding structures called biotrophic interfacial complexes (Oliveira-Garcia and Valent, 2015), whereas some, like *Zymoseptoria tritici* and *Cladosporium fulvum*, spend their entire life cycle extracellularly within the apoplast. Vascular wilt pathogens occur in close proximity to parenchyma cells and colonize xylem vessels (Stotz et al., 2014).

### 2.2 Recognition of fungal virulence effectors

**Figure 3:**
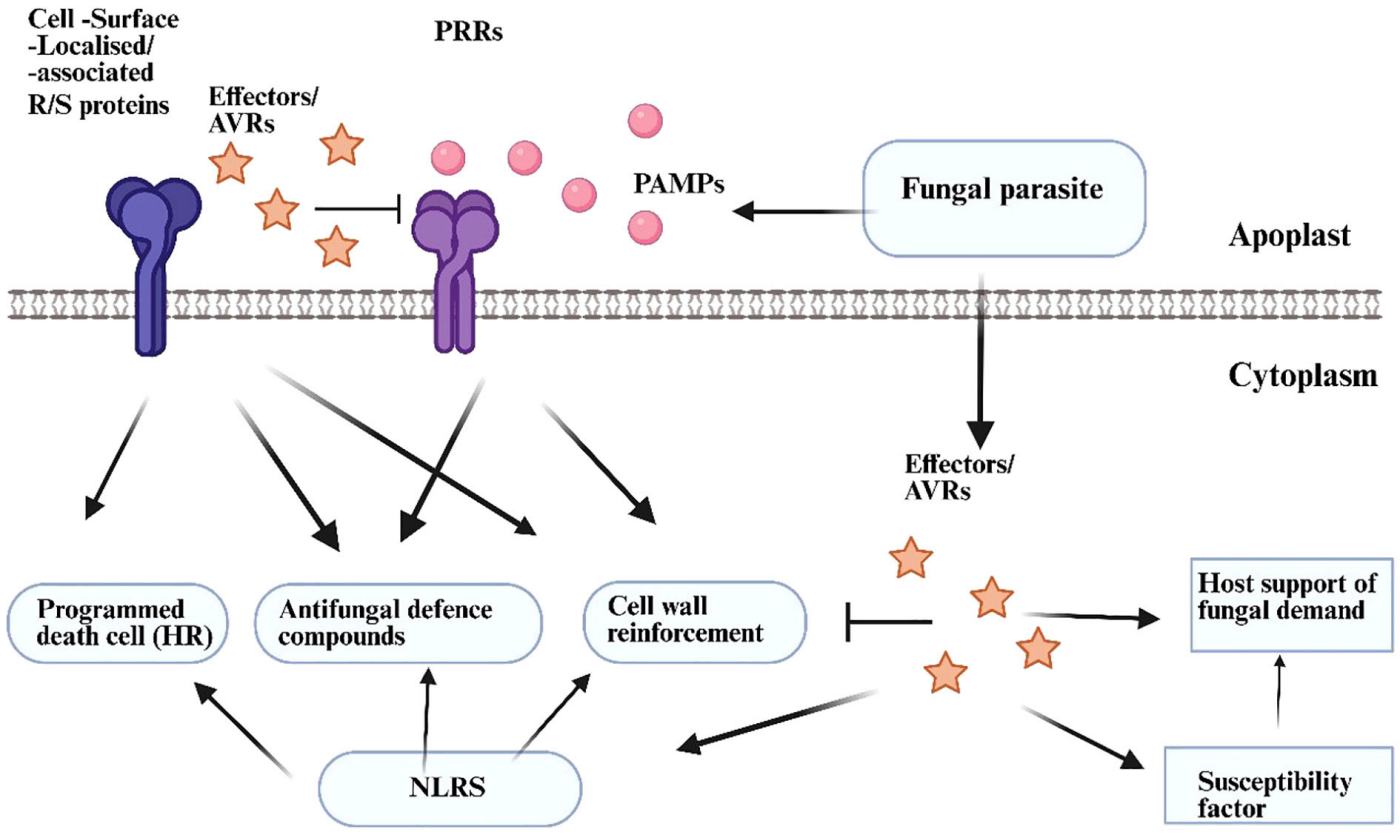
Primary factors influencing plant resistance or vulnerability to parasitic fungus. Plants employ many ways to counteract fungal invaders. Surface-localised pattern recognition receptors (PRRs) recognise conserved microbe-/pathogen-associated molecular patterns (M/PAMPs) emitted from the fungal parasite. This identification triggers defence processes that include cell wall fortification and the synthesis of antifungal defence chemicals. Modified fungi disrupt PRR signalling by secreting virulence effectors into the plant apoplast or cellular interior, hence fulfilling parasitic needs without necessitating defensive suppression. Consequently, immune complexes comprising resistance proteins (R proteins, specifically cell surface R proteins or NLRs) enable resistant plants to selectively identify effectors in the apoplast or intracellularly, where R proteins enhance defence signalling, frequently linked to a hypersensitive response (HR), a specialised variant of programmed cell death. Fungal effectors may activate variables and pathways that serve the pathogen’s needs rather than regulate host immunity. This activation may encompass host susceptibility variables (Saur and Huckelhoven, 2021).

Fungal pathogens employ effector molecules to exploit host activities for their advantage. The predominant category of identified effectors is likely effector proteins; however, small RNAs and metabolites are also released during host infection, and these molecules have demonstrated the ability to function as virulence factors as well (Saur and Huckelhoven, 2021). The majority of effectors are either proteins or metabolites, with fungal effectors occasionally referred to as toxins. The molecular functions of various fungal effectors within the host apoplast have been thoroughly delineated (He et al., 2020), and the molecular principles governing plant-microbe interactions indicate that certain effectors specifically target the microbial recognition machinery of the host, acting downstream of D/M/PAMP recognition, hence disrupting PTI (Dodds and Rathjen, 2010). The localized host cell death, referred to as the hypersentive response (HR), occur during infection by obligate biotrophic pathogens or upon the infiltration of biotrophic elicitors into host tissues, typically resulting from effector recognition by host immune receptors encoded by Resistance (R) genes (Cui et al., 2015; Dodds and Rathjen, 2010). The HR is likely significantly involved in thwarting the invasion of biotrophs, as these pathogens necessitate living host cells for proliferation (Saur and Huckelhoven, 2021). The eradication of fungal infection through R protein function is termed as race-specific resistance, also known as effector-triggered immunity (ETI) in **Figure 3**. The genetic concept under lying ETI was initially articulated by Harold Flor, who explored the genetic basis of flax (*Linum usitatissimum*) resistance to the flax rust fungus *Melampsora lini* (Betzen, 2020). Flor’s research culminated in the formulation of the gene-for-gene theory, which posits that resistance to a particular pathogen strain is governed by a R gene in the plant and a corresponding Avirulence (Avr) gene in the pathogen (Saur and Huckelhoven, 2021). It is evident that Avr genes typically produce effector proteins, which are secreted (**Figure 3**) by pathogens to facilitate pathogen growth in susceptible hosts, such as to circumvent basic host immune responses (i.e. PTI) (Saur and Huckelhoven, 2021).

## 3 Major types of fungal effectors

Plant pathogens receive their classifications through analysis of their nutritional behaviors (Abera Gebrie, 2016). Plant fungal infections pose severe dangers to both the worldwide food supply system and environment stability. Plant pathogenic fungi are classed as biotrophs, hemi-biotrophs, or necrotrophs according to their lifestyle (Wang et al., 2014). Growth patterns of biotrophic pathogens involve obtaining nutrients from living host cells and tissues through low-level excretion of cell wall-degrading enzymes and effectors to suppress host immune responses (Dean et al., 2012). Necrotrophic pathogens pursue growth on the dead host tissues by degrading them before or during colonization period; to provoke cell necrosis, they frequently excrete phytotoxic secondary metabolites (SMs) and peptides, and generate reactive oxygen species (ROS) (Horbach et al., 2011). Hemi-biotrophic pathogens exhibit a biotrophic phase initially during infection, transitioning to a necrotrophic phase thereafter; these pathogens generate toxins exclusively at the final stages to eliminate host cells and complete their life cycle on necrotic tissues (Horbach et al., 2011; Wang et al., 2014).

### 3.1 Biotrophic fungal effectors

Plant pathogenic biotrophic fungi can proliferate within living plant tissue by secreting effector proteins that modify the plant cells’ responses to pathogens and extract nutrition from necrotic or dying plant cells (Gan et al., 2010). Biotrophic fungi have adapted to inhabit live plant tissues and derive nutrients from viable host cells without activating host defenses. The relative impact of the biotrophic lifestyle on the pathogen lifecycle varies among different species. Obligate biotrophs, including rusts and powdery mildews, only develop on living host tissue. Facultative biotrophs, exemplified as *Ustilago maydis*, typically proliferate within living host tissue but are also capable of *in vitro* cultivation (Gan et al., 2010). Biotrophic fungal pathogens of plants rank among the ten most significant pathogens globally in the field of plant pathology (Dean et al., 2012; Mapuranga et al., 2022). In biotrophic infections, the fungus generally proliferates within plant cells, as defined by the cell wall, yet remains isolated from the host cytoplasm by the plant plasma membrane. Intracellular growth may manifest as invasive hyphae or as specialized feeding structures termed haustoria, exemplified by rust fungus and powdery mildews (Gan et al., 2010). Biotrophic fungi release effector proteins that alter host cellular processes, inhibit immunological responses, and disrupt hormone signaling pathways. For example, the effectors primarily target components of salicylic acid (SA) pathway (Bauters et al., 2021; Leiva-Mora et al., 2024) and jasmonic acid (JA) pathway (Leiva-Mora et al., 2024; Tariqjaveed et al., 2021). The redox environment of plants gets modified by Biotrophic fungi through its control of antioxidant enzymes which leads to decreased defense responses and better conditions for colonization (Leiva-Mora et al., 2024; Park and Son, 2024). Biotrophic pathogens must manipulate host physiological processes to extract nutrients from living host cells and tissues for survival and life cycle completion; thus, they secrete effectors to inhibit host immunity while minimizing host cell damage to facilitate colonization of living cells (Laluk and Mengiste, 2010; Lo Presti et al., 2015; Zhang et al., 2022).

### 3.2 Hemibiotrophical fungal effectors

Hemibiotrophic organisms start by using living tissue before continuing their development as necrotrophic colonizers of dead tissues (Giraldo and Valent, 2013). Organisms that only possess a limited host variety begin their process by living off the plant cells before their life cycle ends with necrotrophic plant tissue destruction for nutrient extraction. Throughout their biotrophic developmental stage several species produce haustoria along with appressoria but subsequently produce hydrolytic enzymes and toxins during the necrotrophic stage (De Silva, 2016). Hemibiotrophic fungal pathogens need specific effectors because they require these effectors throughout biotrophic and necrotrophic phases to progress infections. Hemibiotrophs employ two distinct types of effectors to promote cell death during later infection stages and regulate the biotrophy-necrotrophy switch (BNS) (Shao et al., 2021). Hemibiotrophic pathogens start with biotrophic infection before turning necrotrophic after which the biotrophic phase lasts between single cells to multiple cells within various pathosystems (O'Connell et al., 2012).

### 3.3 Necrotrophic fungal effectors

The definition of necrotrophs describes microorganisms which obtain their nutrients from migrated host cells using mechanisms that lead to cell death. Necrotrophic fungi use effectors to activate host programmed cell death (PCD). Multiple documented studies investigate how necrotrophic fungal effectors lead to host cell death. Research on fungal proteins that provide tolerance to specific conditions requires an analysis of plant death processes because phytochemicals produced during death may harm fungi. Detailed information in the detoxifying mechanisms can be found in a recent review concerning necrotrophic fungus (Westrick et al., 2021).

**Figure 4:**
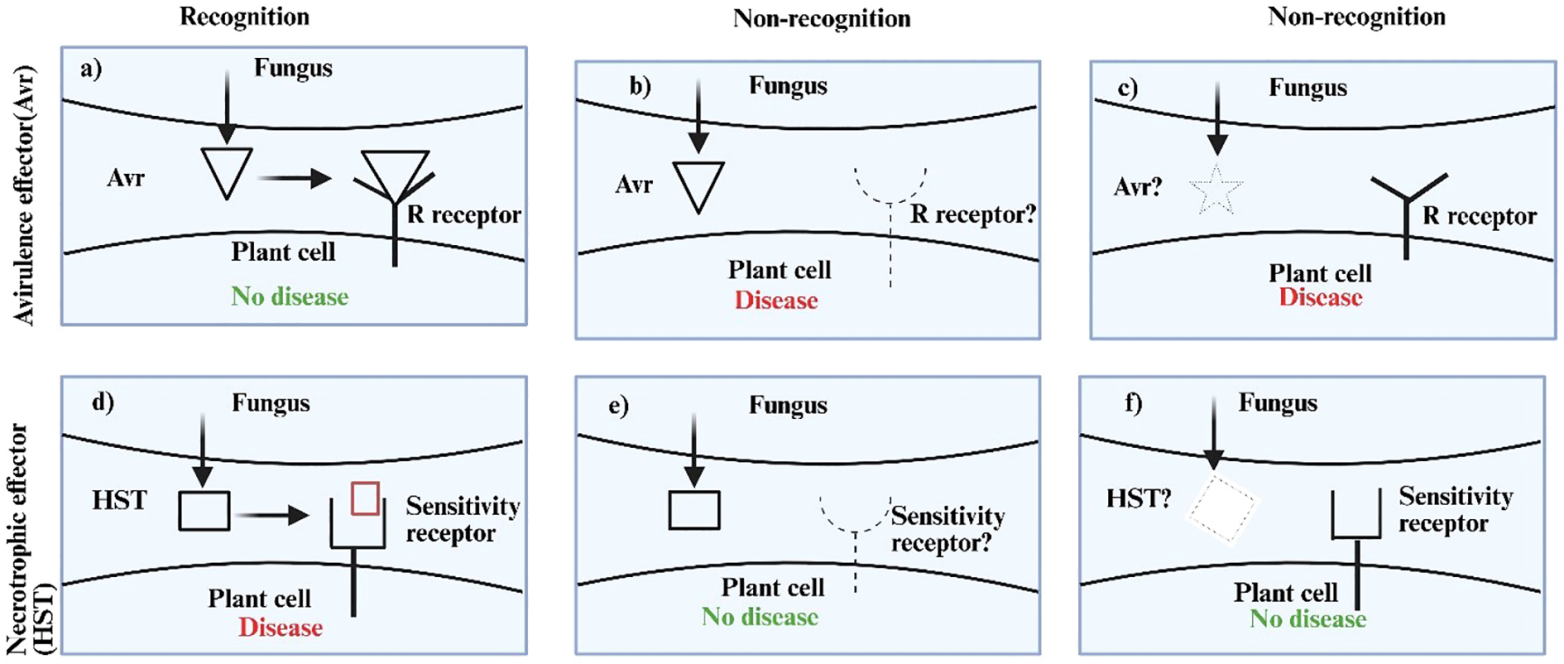
Outcomes of fungal effector–host interactions. **(a)** to **(c)** illustrate the host reaction to a fungal Avr protein. The host will develop an effective defensive response against the virus if Avr recognition takes place **(a)**. Infection will ensue if the pathogen is unrecognised due to a deficient or modified R (resistance) receptor **(b)** or Avr effector **(c)**. Necrotrophic effectors work in an inverse manner **(d–f)**. An effective infection will transpire just during effector recognition **(d)**. In the absence of recognition, no disease will occur due to a missing or modified host sensitivity receptor **(e)** or fungal effector **(f)**. Receptors are depicted on the cell membrane for clarity. We acknowledge that some host receptors are situated intracellularly (Tan et al., 2010).

The hypersensitive response (HR) is a localized cell death mechanism initiated by the detection of effectors by plant resistance (R) proteins, providing resistance against biotrophs and hemibiotrophs, and is commonly known as Effector Triggered Immunity(ETI) (Shao et al., 2021). This genetic program can be exploited by necrotrophs for their own advantage, given their trophic lifestyle. This condition is referred to as effector-triggered susceptibility (ETS), characterized by the activation of plant defense mechanisms, frequently resulting in cell death and increased vulnerability to necrotrophic pathogens (Williams and Dickman, 2008). These necrotrophic effectors were originally designated as “host selective toxins” (HST) and are often effective within a limited spectrum of plant hosts [45]. Necrotrophic effectors have certain similarities with the avirulence (Avr) effectors of biotrophic fungal pathogens (**Table 1**). In contrast to the standard gene-for-gene concept, which posits that the interaction of avirulence effectors with host resistance (R)-gene complexes results in resistance, necrotrophic effectors operate in an ‘inverse’ manner. An interaction between a necrotrophic effector and the product of a dominant sensitivity gene in the host results in disease (Tan et al., 2010) (**Figure 4**).

**Table 1:** Properties of proteinaceous avirulence and necrotrophic effectors.

Characteristic	Avirulence effector	Necrotrophic effector
Relative small size A	Yes	Yes
Secreted	Yes (Some may be anchor to or be located at the hyphal cell wall)	Yes (Some may be anchor to or be located at the hyphal cell wall)
Location of recognition	Intra- or extracellular	Unknown
Cysteine-rich B	Predominantly	Some
Compatible host response	No disease	Disease
Fungal lifestyle	Biotrophic or hemibiotrophic	Necrotrophic
Function during host recognition	Hypersensitive reaction that results in the containment of the infection, Either by indirect Avr recognition (guard proteins) or direct Avr recognition (cognate resistance proteins).	Necrosis of the tissue caused by host cell death. Effector recognition by the host’s dominant sensitivity proteins.
Role in virulence	Some Avr proteins, though largely unknown, function as protease inhibitors and bind chitin, providing protection against plant chitinases.	While mostly unrecognized, certain effectors influence the host’s photosystem and plasma H^+^ ATPase activities.

A = Under 30 kDa. B = Greater than four cysteines per mature polypeptide (Tan et al., 2010).

## 4 The subtle distinction between necrotrophs and heminiotrophs

The main differentiating factor between hemibiotrophs and necrotrophs rests on their ability to form haustoria or haustoria-like structures from intracellular hyphae which makes hemibiotrophs similar to biotrophic pathogens (**Table 2**). During the pathogenic process hemibiotrophs produce a bulged intracellular hypha first which is enclosed by the host plasma membrane then transforms into a flatter intracellular hypha resulting in necrosis [66]. Necrotrophic fungi show extracellular hyphal growth that does not include intracellular multiplication while they gain access through stomatal apertures or hydathodes. Both hemibiotrophic and necrotrophic lifestyles encompass an early biotrophic phase succeeded by necrosis. The duration of the biotrophic phase exhibits variability among hemibiotrophs and may exhibit even greater variability in necrotrophs. This duration can be influenced by numerous external environmental factors. These necrotrophic pathogens can create either appressoria or appressoria-like structures (ALS) while some also produce bulbous hyphae that resemble structures observed in hemibiotrophic pathogens [67].

**Table 2:** Main features of necrotrophic and biotrophic/hemibiotrophic fungi and dominant plant defense pathways.

Plant and pathogen features	Necrotroph	Biotroph/Hemibiotroph	References
Dominant immune signaling pathway	Generalist necrotrophs: PTI Host-specific necrotrophs: HST detoxification or insensitive target	ETI, PTI	(Jones and Dangl, 2006)
Major defense hormone	Jasmonate and ethylene	Salicylate	(Pieterse et al., 2012; Durrant and Dong, 2004)
Major immune elicitors	Chitin, cutin, OGs, endogenous plant peptides (phytocytokines)	Chitin, some fungal-derived short peptides(e.g. hemibiotrophic effectors)	(Boller and Felix, 2009; Galletti et al., 2008; Pieterse et al., 2014)
Effector- NLR interaction	Effector-triggered susceptibility	ETI, hypersensitive response	(Dodds and Rathjen, 2010; Oliver and Ipcho, 2004)
Main effectors and disease agents	Toxins, CDIPs, secondary metabolites, CWDEs	Avr effectors, largely proteins	(Hogenhout et al., 2009; Möbius and Hertweck, 2009)
Major immune responses or resistance factors	Complex, cell death inhibition, some PR proteins, secondary metabolites, toxin detoxifying enzymes	HR, ROS, PR proteins	(Torres et al., 2006; van der Does and Rep, 2017)
Pathogen infection strategy	CWDEs, toxins, and digest plant cell wall and colonize tissues	Develop infection structures and use different techniques for nutrition intake and host tissue penetration.	(Mendgen and Hahn, 2002; Oliver and Ipcho, 2004)
Cell death (early during infection)	Susceptibility	Resistance	(Greenberg and Yao, 2004; van Kan, 2006)
Fungal infection structures	Generally, less differentiated and simple structures	Some exhibit simple structures, while others have highly complex and differentiated features.	(Mendgen and Hahn, 2002; Oliver and Ipcho, 2004)
Major disease symptoms	Decay, maceration, rots, molds	Mild cell damage, Plant cells remain alive throughout the infection	(Glazebrook, 2005; van Kan, 2006)

ETI, Effector-triggered immunity; PTI, PAMP-triggered immunity; HST, Host-specific toxin; OG, Oligogalacturonide; NLR, Nucleotide-binding domain leucine-rich repeat; CDIP, Cell death-inducing protein; CWDE, Cell wall degrading enzyme; HR, Hypersensitive response; ROS, Reactive oxygen species (Liao et al., 2022).

**Figure 5:**
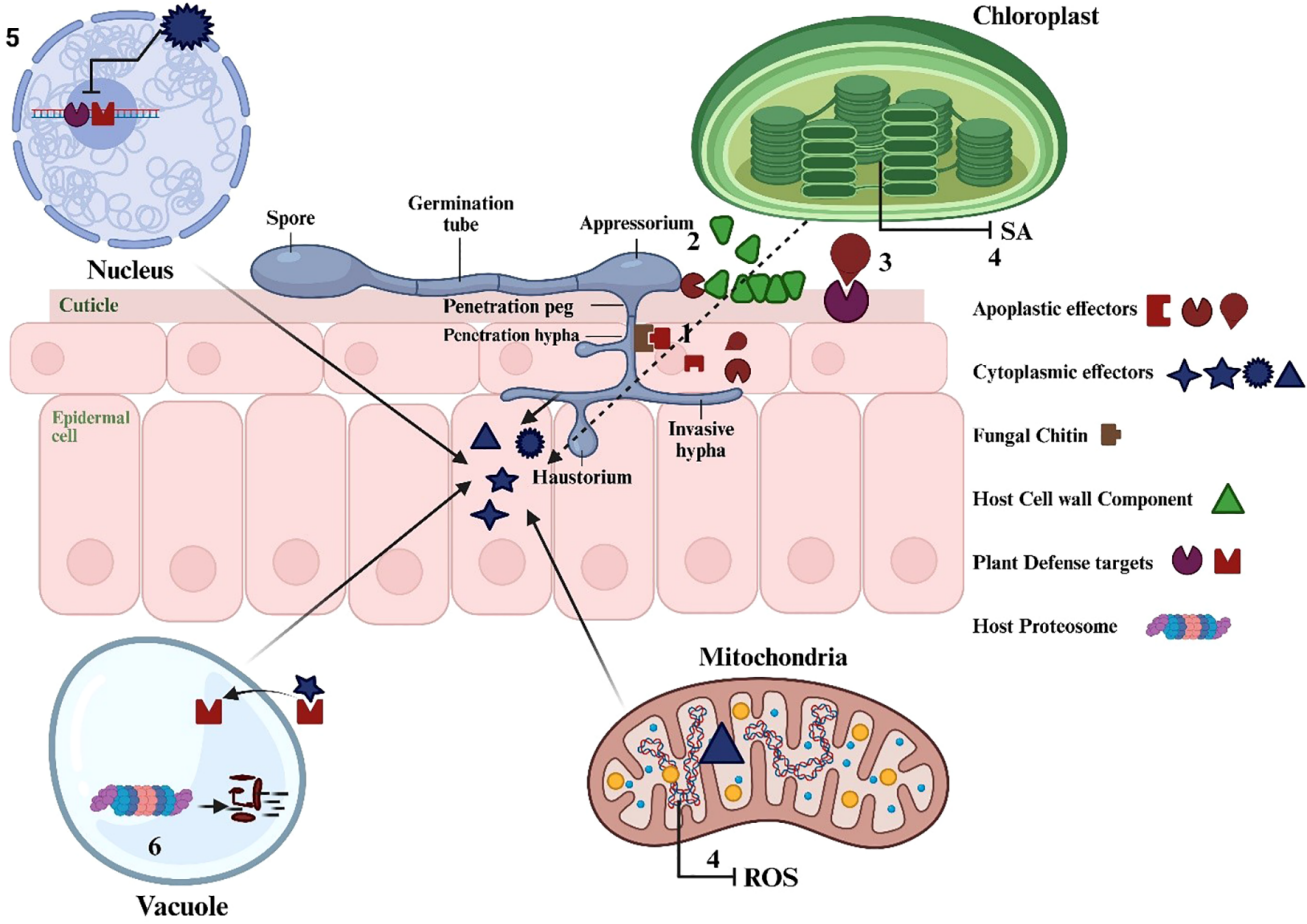
Spaces and structures that form the apoplast in plants (Farvardin et al., 2020).

Apoplastic: E. Münch initially employed the term “apoplast” in his 1930 German scientific publication (Farvardin et al., 2020). The two fundamental elements of apoplast include intercellular spaces that comprise gas and water between cell membranes and the fibrils and micelles containing substances within cell walls and xylem structures. Plants possess the rhizoplane and cuticle areas and these regions together form an extension of the apoplast system (**Figure 5**) (Farvardin et al., 2020). Filamentous microorganisms establish relationships with plants that vary from mutualistic (fungi) to pathogenic (fungi and oomycetes).The result of these interactions, whether compatibility or incompatibility, is frequently established in the apoplast, where the initial contact between microbial and plant cells occurs (Doehlemann and Hemetsberger, 2013; Rocafort et al., 2020). The apoplast constitutes a hostile environment, characterized by the constitutive production of proteases, protease inhibitors, secondary metabolites, and hydrolytic enzymes by plants to limit fungal and oomycete growth (Rocafort et al., 2020).Cell surface-localized immune receptors monitor the apoplast and recognize invasion patterns, activating the plant immune system (Cook et al., 2015; van der Burgh and Joosten, 2019). This activation inhibits or ceases fungal and oomycete proliferation by generating supplementary defensive chemicals and reactive oxygen species (ROS), with callose and lignin deposition, and, in certain instances, the hypersensitive response (HR). The apoplast is not a sterile environment; it contains various bacteria that struggle for space and nutrition (Rovenich et al., 2014; Snelders et al., 2018). These microorganisms utilize hydrolytic enzymes, antibiotics, toxins, and volatile compounds that can further inhibit the growth of fungi and oomycetes (Carrión et al., 2019).

Apoplastic effectors are released into the interfacial area between pathogen and plant cells, where “chemical warfare” transpires (Doehlemann and Hemetsberger, 2013; Harris et al., 2023). These effectors comprise protease inhibitors (Rooney et al., 2005) detoxification of secondary metabolites (Okmen et al., 2013; Singh et al., 2023), chitinase- binding effectors (van den Burg et al., 2006), and peroxidase inhibitors (Hemetsberger et al., 2012).These effectors concentrate on mitigating the pathogen’s surroundings and evading detection (Harris et al., 2023).

**Figure 6:**
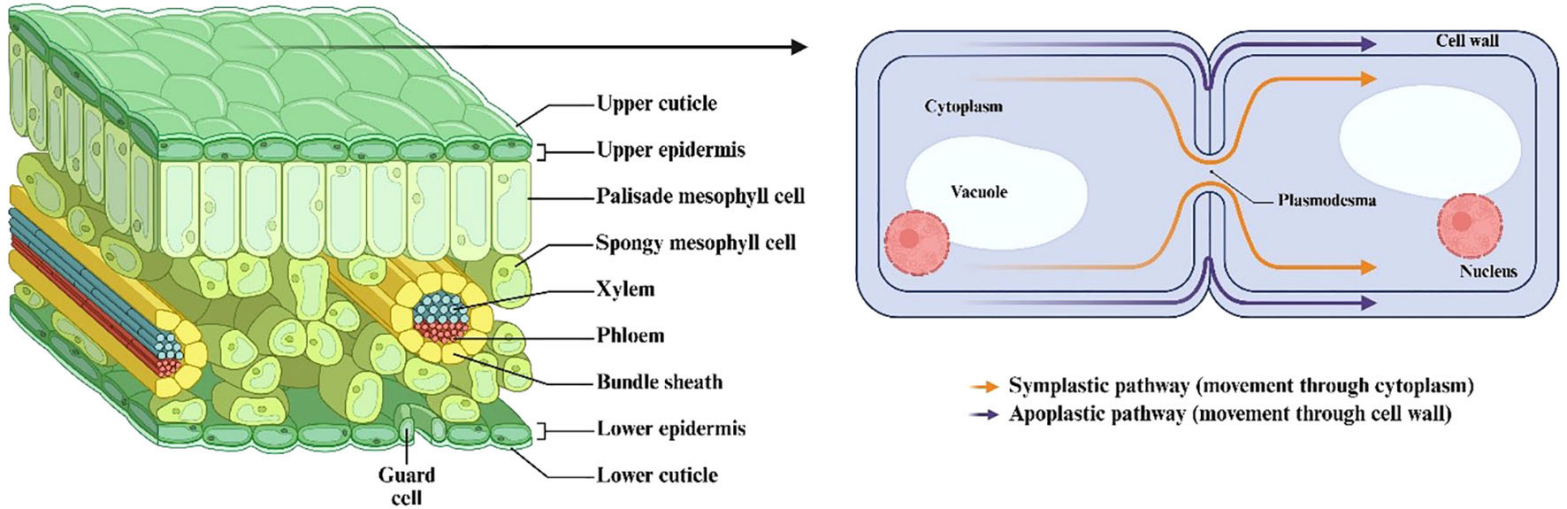
A plant cell illustrating the conserved host colonisation methods of fungi. A fungal hypha is illustrated colonising the apoplastic space of a plant cell, showcasing examples of apoplastic and cytoplasmic secreted effectors together with their respective host targets. Apoplastic effectors may serve to (1) circumvent chitin-induced plant immune recognition, (2) breakdown the plant cell wall, or (3) interact with host proteins to modify the microenvironment, alter host defences, and facilitate colonisation. Cytoplasmic effectors can localise to subcellular compartments to (4) disrupt defence signalling pathways via mitochondria or chloroplasts, (5) reprogram transcription, or (6) target or imitate host proteasome machinery to modulate plant immune responses (Li et al., 2024).

Cytoplasmic effector: Effectors are classified as cytoplasmic when they are translocated into the host cell to target host cellular processes (Dulal and Wilson, 2024). Cytoplasmic effectors are secreted proteins that facilitate fungal disease by targeting plant organelles and altering host defenses (Li et al., 2024; Sperschneider and Dodds, 2022; Zeilinger et al., 2016). They function within the host’s intracellular space, affecting numerous cellular processes and generally have a greater ratio of positively charged amino acids (Li et al., 2024; Sperschneider and Dodds, 2022; Zeilinger et al., 2016). ​ Fungal pathogens that utilize these effectors can severely impact forests by causing tree mortality, diminishing plant species diversity, and facilitating infection (Bi et al., 2021). Cytoplasmic effectors typically facilitate fungal disease by targeting plant organelles to alter various cellular processes within the host (Bi et al., 2021; Li et al., 2024; Sperschneider and Dodds, 2022). Cytoplasmic effectors are essential in fungal pathogenesis as they inhibit plant defense mechanisms and modify host cell structure and function. They enable pathogen colonization and alter host defenses to enhance infection (**Figure 6**). For example, The BcCrh1 protein from *Botrytis cinerea* serves as a cytoplasmic effector and a trigger of plant defense mechanisms. Numerous effector-encoding genes are organized in clusters and are variably activated when various plant tissues are colonized (Bi et al., 2021; Li et al., 2024; Sperschneider and Dodds, 2022).

## 5 Characteristics of effectors

Effectors represent small molecules released by microbes that transform cell structures of their hosts to make pathogen-host communication possible (Fabro, 2022; Langin et al., 2020). The molecules produce physical along with physiological alterations within the target species while also influencing the producing microorganisms in select cases (Cai et al., 2023; Figueroa et al., 2021; He et al., 2020). Most effectors are proteins (Carreon-Anguiano et al., 2020; Sperschneider and Dodds, 2022), though some are secondary metabolites (Rangel and Bolton, 2022), or small RNAs (Yamankurt et al., 2020). The absence of conservation across different organisms among known effectors led to the creation of *in silico* predictive identification methods which use broader structural criteria. At least four screening criteria exist for potential phytopathogen effectors where (a) amino acid count remains lower than 400 amino acids, (b) signal peptides facilitate the protein’s escape from cellular secretion by phytopathogen cells (Carreon-Anguiano et al., 2020; Sperschneider et al., 2018), c) the amount of cysteine amino acid reflects effector enrichment and; and (d) transmembrane domains must be absent in candidates (Carreon-Anguiano et al., 2020). The identification process for such effectors becomes more streamlined because their specific expression patterns become evident when the phytopathogen interacts with its host (Tao et al., 2020; Toruno et al., 2016). The amino acid sequences of protein effectors in oomycetes often show recurring small sequence patterns named motifs which include RxLR, CHXC or LFLAK (Fabro, 2022). Some microbial effectors have their genetic locations on dispensable chromosomes which may be missing from certain microbial strains together with regions of the chromosome that contain repetitions and lack high gene density (Peng et al., 2019). A study analyzed the location of virulence-related genes in fungus *Pseudocercospora fijiensis* which revealed most genes existed in “dispensable” genomic regions while transcriptome analysis showed these genes become active when *P. fijiensis* infects banana (*Musa acuminata*) (Noar and Daub, 2016), the *P. fijiensis* effectors were predicted through EffHunter software application using effector characteristics (Carreon-Anguiano et al., 2020), The analysis discovered 136 effectors which reside within dispensable genomic regions as well as genomic areas common to all strains designated the “core” genome (Todd et al., 2023). The evolution of effector protein genes encounters high selection pressure because of which their mutation rates surpass other gene families (Todd et al., 2023). Shared effectors between strains of the same species commonly develop polymorphisms because of host adaptation together with virulence expression (Kanja and Hammond-Kosack, 2020). Research shows that effectors exist in both closely related microbial populations as well as only in distantly related microbial groups (Todd et al., 2023). Orthologous proteins show minimal sequence similarity because they share functional identities with other proteins throughout different animal groups although their sequences have numerous variations which originated from their common ancestral origins (Todd et al., 2023). Avr4 from *P.fijiensis* shares only 50.5% sequence similarity with its corresponding ortholog in *Cladosporium fulvum* while these species both belong to the Dothidiomycetes fungal class (Todd et al., 2023). Latest omics studies detect effector protein domains and motifs even though most effectors display minimal sequence identity between family members (Todd et al., 2023). Different recognized domains exist with LysM and ceratoplatanin and RNAase while necrosis-inducing protein domains (NPP1 or NEP) and CFEM also belong to this classification (Carreon-Anguiano et al., 2022; Outram et al., 2021; Zhou and Zhang, 2020). Modern research demonstrates how microorganisms carry hundreds of effectors (Carreon-Anguiano et al., 2020; Noar and Daub, 2016; Sperschneider et al., 2018), which work at different times (Noar and Daub, 2016; Toruno et al., 2016). Plant effectors function to interrupt essential signalling procedures and phytoregulator production as well as plant defensive systems (Fabro, 2022; Han and Kahmann, 2019; Plett et al., 2020).

## 6 Function of effectors

All plant-microbe interactions depend on effectors since these molecules help phytopathogens cause damage while promoting beneficial relationships with helpful microorganisms including mycorrhizae (Plett et al., 2020; Todd et al., 2023), and new studies investigate their function during microbe-microbe interactions (Snelders et al., 2021, 2020). The infection approach of phytopathogens determines the functioning of their effectors because biotrophic pathogens need living hosts to succeed but necrotrophic pathogens obtain nutrients by utilizing dead tissue. The infection pattern of hemibiotrophic phytopathogens spans two nutrient acquisition phases starting with survival in live tissue then continuing in deceased tissue cells (Todd et al., 2023). Biotrophic organism effectors block the host immune response while necrotrophic effectors generate excessive non-localized defenses that eventually destroy their host cells (Todd et al., 2023). The effectors of hemibiotrophic phytopathogens work to delay cellular demise at first but they launch effectors in the necrotrophic stage that advance host mortality (Castillo-Sanmiguel et al., 2022; Jones and Dangl, 2006; Thordal-Christensen, 2020; Todd et al., 2022b). For example, the effector BcNEP1 exhibits high levels of expression during the initial stages of *Botrytis cinerea* infection but the effectors BcSSP2 and BcNEP2 become active only after the early phase (Zhu et al., 2022). In the case of *Colletotrichum* spp. hemibiotrophic fungi effectors have specific functions during the biotrophic stage but induce death of host cells to move into necrotrophic development (Ono et al., 2020; Tsushima et al., 2021). Until the last decade researchers understood all effectors to be extracellular which led to their inclusion in the definition of effectors but the most extensively studied ones are apoplastic (extracellular) (Carreon-Anguiano et al., 2020). The field of effectoromics now recognizes intracellular effectors which function either inside the cytoplasm or the organelles as prominent new discoveries (Sperschneider and Dodds, 2022; Tariqjaveed et al., 2021; Todd et al., 2022a). Apoplastic effectors include small protein molecules that work through enzymatic degradation of cell walls combined with exploits that loosen these structures and additional activities such as protease inhibitory functions and inability for plant recognition of pathogenic organisms (Fabro, 2022; He et al., 2020; Langin et al., 2020). The biological functions together with the cellular locations of intracellular effectors within host cells demonstrate variability because most targets are essential defense proteins in plant immunity (Thordal-Christensen, 2020). Several targets encompass proteases and components from the ubiquitin and proteasome systems with addition to transcription-related proteins and receptors and biosynthetic machineries and phytoregulator-regulated signaling pathways that control defense responses in plants (Fabro, 2022; Han and Kahmann, 2019).

Microorganisms use their effector proteins to manipulate jasmonate (JA) and salicylate (SA) and ethylene (ET) phytoregulator production processes for their own benefit (Alhoraibi et al., 2019; Chini et al., 2018; Langin et al., 2020). For example, the fungal effector Cmu from *Erysiphe quercicola* functions as a chorismate mutase to deteriorate host salicylic acid synthesis (He et al., 2021). The VdIsc1 effector produced by *Verticillium dahliae* displays isochorismatase activity to block salicylic acid synthesis just like the fungus (Zhu et al., 2017). Through its function as RipAB *Ralstonia solanacearum* blocks the signaling mechanisms controlled by salicylic acid (Qi et al., 2022). Effectors produced by microorganisms serve two functions by maximizing phytoregulator synthesis or synthesizing close analogues of phytoregulators (Todd et al., 2023). For example, *Lasiodiplodia mediterranea* produces lasiojasmonate A (LasA) which acts as an analogue of jasmonic acid during necrotrophic infection of host plants. The pathogen uses LasA to produce the strong jasmonic acid regulator jasmonyl-isoleucine (JA-Ile) which boosts signaling mechanisms leading to cell death during necrotrophic development (Chini et al., 2018).

## 7 Tools for studying fungal effectors

- Identification: The identification of effectors and the prediction of their localisation are advancing due to the continuous enhancement of machine learning models trained on experimentally validated apoplastic and cytoplasmic effectors (Sperschneider and Dodds, 2022). Functional prediction can be further applied to sequences that are unrelated but structurally identical effectors based on common protein structural folds (Jumper et al., 2021; Seong and Krasileva, 2023). Identifying new effectors by their fast evolution via host and pathogen interaction networks is another effective strategy (Sugihara et al., 2023).
- Functional importance: Comprehending the biological role of an effector necessitates experimental varification. Reverse genetics methods, such as RNA silencing and CRISPR knockout, are frequently used to evaluate the direct involvement of an effector in a specific interaction (Li et al., 2024). Simultaneously, heterologous expression systems that transiently express effectors in non-native hosts have also been established as adaptable tools, particularly when genetic engineering strategies are impractical or when a swift and systematic screening of interactions between effector proteins and host is required (Lee et al., 2012). The induction or suppression of specific plant immune responses suggests the potential role of the candidate effector in the host–fungi interaction, while the presence or absence of the signal peptide can intentionally guide the effectors into either apoplastic or cytoplasmic space, respectively (Tintor et al., 2020).
- Interactive partners: Numerous effectors modify host processes through interactions with host proteins, and there is a growing array of techniques to discover effector targets. Protein constructs such as GFP, RFP, and tdTomato, are utilized to verify the subcellular localisation of cytoplasmic effectors (Park et al., 2017). Yeast two hybridization and co-immunoprecipitation succeeded by liquid chromatography-mass spectrometry are established methodologies for identifying effector–partner complexes (Petre et al., 2021). The turbo biotin ligase tag (TurboID) facilitates *in vivo* proximity labelling and co-immunoprecipitation (Shi et al., 2023).

## 8 Conclusion

Forest ecosystems rely on fungal pathogens because these organisms facilitate natural decomposition yet pose an important threat as they cause plant ecosystem damage and biodiversity decline through invasions. Multiple effector systems of pathogens operate to alter plant defensive mechanisms during infection so they can spread infection more effectively while supplementing nutrient requirements. The microbial effectors launch attacks on plant protective defenses to initiate an extended evolutionary conflict between pathogen agents and plant organisms.

Fungal groups establish different life paths that assign them to biotrophic, necrotrophic and hemibiotrophic pathways in their interactions with host organisms. The living conditions of biotrophic fungi enable them to occupy plant cells yet necrotrophic fungi survive through cellular death of their hosts. Pathogens employ multiple strategies for activating plant defense mechanisms which proves that plant-pathogen systems function as well-adapted complex systems.

The virulence of pathogens increases because effectors both alter plant metabolic operations and signaling pathways and immune response functions and directly regulate phytohormones to block defense mechanisms. Host-specific behavior together with pathogen survival develops through effectors that adapt because of high mutation rates and evolutionary pressures. Studies of genetics show that effector genes find shelter within mobile genomic zones because these mobile areas produce specialized and varied features. Sustainable plant disease control methods need extensive identification of molecular plant-pathogen interactions to protect forest ecosystems. Research efforts need advancement regarding fungal effectors and plant immune responses and cytoplast and apoplast microbial communities because this information supports sustainable forest management and plant pathology research.
